# Experiments on Kiesteine, with Observations on Its Application to the Diagnosis of Pregnancy

**Published:** 1842-09

**Authors:** Elisha K. Kane


*Experiments on Kiesteine, with observations on its application to the diagnosis of pregnancy.* By Elisha K. Kane, M. D. The following extracts are from a valuable paper under the above title, in the last number of the *American Journal of the Medical Sciences.* The paper was an inaugural dissertation for the degree of Doctor of Medicine, and is published in accordance with the recommendation of the Medical Faculty of the University of Pennsylvania.

"My mode of conducting the experiments was this. The recent urine was placed in open glass cylinders, of diameters varying from an inch and a half to that of a common tumbler, and protected from the dust by paper covers. Those were arranged in a dry, well ventilated room, where the temperature was uniform and moderate, and were exposed in groups to the equal action of air and light. I examined them frequently during the day; but as the changes were not rapid, I determined after a little while to note only one set of observations in the twenty-four hours. My notes were made upon the spot. If from any cause, an individual observation or a series was unsatisfactory, or inconclusive; or if it led to a different result from others, I repeated it at once with increased care; and I was always careful to observe the constitution, habits and circumstances generally of each patient.

The examination of the first group of cases satisfied me, that the urine during pregnancy assumes appearances different from those witnessed under other circumstances, and which I was therefore disposed to regard as characteristic of that state. Subsequent inquiries confirmed me in the general accuracy of this opinion, but compelled me at the same time to admit its liability to exception.

The more obvious of these appearances regard the superficial formation described by Dr. Bird, and recently investigated by Drs. McPheeters and Perry; but there are others which point to a series of intestine changes somewhat more obscure, though scarcely less interesting. My observations applied to both; and I regret that the limited time at my command during the studies preliminary to graduation, obliges me to select a single class as the subject of this dissertation. I take, however, that indication which seems to be best fitted for practical usefulness in diagnosis, the *pellicular change*, and which I suppose to be most properly called the Kiesteine.

The urine, submitted to observation in the way I have described, presents but little change during the first thirty-six hours. The mucous flocculi, if they exist, gradually subside during this period, forming a whitish cloudlike deposite at the bottom and sometimes on the sides of the glass; while more or less alteration occurs in the color and tranparency of the fluid.

The surface remains for a short time entirely unchanged; but in most cases, a greater or less number of shining acicular specks, apparently crystalline, begins to be seen within the first eighteen or twenty-four hours. These are generally scattered over the surface without regularity; but in some rare cases, they are so disposed as to form a translucent film of uniform thickness, which afterwards assumes the more defined characters of the pellicle. How far these crystals are essentially connected with the formation of the pellicle, I am not prepared to say. In many cases, I have not succeeded in detecting their presence, even by a microscope; and, indeed, I have failed to discover any *unvarying* indications whatever of the approaching development of the kiesteine.

The cloudlike appearance, which is alluded to by Nauche and Eguisier, although possessed of much interest, I have not found to be a uniform premonitor of the forming pellicle; I have supposed it to be nothing more than the Enæorema of the older writers, depending upon the imperfect aggregation of mucous flocculi; for I have seen it repeatedly when there was no pregnancy to account for it, and it was uniformly absent where the fluid presented perfect transparency.

The time at which the pellicle begins to form varies considerably. I have seen it well marked at the end of thirty-six hours, and have known it to make its first appearance as late as the eighth day. At first, it is hardly discernible. It is generally seen forming at the centre or on the sides of the glass, presenting a delicate milky or bluish white aspect. It is however in some cases uniformly disposed over the surface from the commencement, and assumes the appearance of a nearly transparent film, which gradually becomes more distinct. But it has not always the continuous strongly marked character, which some have ascribed to it. I have seen it begin in striated irregular lines, somewhat resembling a spider's web, in rings, circles, trapeziums, and irregular figures of almost every shape, which gradually became obscured by the full development of the pellicle.

When it has attained this stage, which occurs generally about the fifth day, it presents a continuous scum of an opaline white or creamy appearance, with a slight tinge of yellow, which gradually becomes deeper and more decided. The uniformity of this color, however, is generally broken by granulated spots of a clearer white, giving it a dotted or roughened aspect. The crystals of the forming stage now appear like shining points, and I have sometimes found numerous small brownish specks, sprinkled over the surface, not unlike the gratings of nutmeg. It is at this period, that the pellicle may be compared "to the fatty scum of cooled broth."

In this state it continues for some time, preserving all its characters unbroken. The glass, where the surface meets it, is discolored by a white opaline ring; and a series of such rings, varying in extent from a line to the fourth of an inch, marks the descent of the surface during the progress of evaporation.

The cheesy odor, mentioned by Dr. Bird as a valuable aid in diagnosis, and as "by no means unfrequent in those specimens in which the pellicle is very thick," I have found in but seven cases. Many pellicles of great thickness were entirely without it; and in two of those presenting it, the pellicle was thin and not very well developed. Drs. McPheeters and Perry were unable to detect it in either of the twenty-seven cases examined by them, and I have found it unequivocally developed in at least three cases in which pregnancy did not exist.

The pellicle, if left undisturbed for some days, breaks into cracks, commencing generally from the central portions, but not always extending to the edge of the glass. These are again crossed by other fissures, and the pellicle is more or less broken up. In the mean time, the flakes, which have been forming from the commencement of disintegration, have their edges depressed into the fluid, while at the same time the general thickness of the pellicle is much diminished; and this depression or dip gradually increasing, the depending particle s detached, and sinks slowly to the bottom. Its complete disintegration, however, is but seldom seen; being anticipated by the decomposition of the fluid. The deposite is of course considerably increased by the fallen portions of the pellicle, and is found irregularly disposed over the bottom of the vessel; but as I have remarked, most abundant on the side farthest from the light.

I cannot agree with those who consider this deposite as presenting well marked distinctive characters to the eye, and I certainly have not found it uniformly coincident with the approach of the pellicle. It has indeed in many cases been absent at that period; and in others, until augmented by the detached pellicle, I have been unable to distinguish it from the very many deposites found in other urine. How far a chemical investigation may give it value, I am not prepared to say: although its liability to be confounded with other sediments makes it practically unavailable as a test, it offers a fine field for microscopic and chemical research.

This description of the appearances and changes of the pellicle, though more detailed than those of Nauche and his followers, still applies only to the better defined examples. In a considerable proportion of cases, some of the phases mentioned are not to be found together; and I have not been able by the most careful observation to discover the causes of variance. This I allude to here, because the absolute and unqualified language, which I have met elsewhere upon the subject, seems to me calculated to mislead the unpractised inquirer.

It must not however be inferred that the presence of kiesteine is determinable only by vague and undefined characteristics. On the contrary, the tables which are appended to this dissertation, will show that they are as well defined as most pathological phenomena, though like them they sometimes require for their discrimination a practised comparative scrutiny.

The table marked A exhibits a condensed record of my observations, more or less frequently repeated, on the urine of eighty-five pregnant females. Of these, as will be seen, sixty-eight gave a well marked pellicle of the sort called kiesteinic, eleven gave the pellicle under a modified form, but with appearances which enabled me to recognize it clearly, and six gave no pellicle whatever. Of these last, one was laboring under mammary abscess and convalescing from typhoid fever, and one was in a condition of extreme anæmia from repeated uterine hemorrhages; but the others, unless they succeeded in practising reiterated deceptions on me, which I can hardly believe, must be regarded as absolute exceptions."

"The urine of unimpregnated females in a state of health rarely undergoes any change which in this respect can be misapprehended. I have examined twenty-eight cases in a perfectly healthy condition, and have sometimes known pellicles form on the urine, as well as on that of males; but the distinctive character of the kiesteine was wanting in every case.

In certain pathological conditions, however, discrimination is somewhat more difficult. The pellicle that is not unfrequently seen on the urine in the last stages of phthisis, in arthritic diseases, and in cases of metastatic abscess, vesical catarrh, and uterine tumours, has points of resemblance to the kiesteinic which might readily mislead the unpractised."

"The particulars in which the kiesteine differs from other pellicles regard the manner of its formation and departure, even more than its appearance when developed. As I have already mentioned, it generally begins to show itself within a day, or at furthest within two days after the discharge of the fluid, and advances gradually to its complete development. The other pellicles, on the contrary, rarely give indications of their approach until the fluid has stood a longer time, or even till decomposition has supervened, and then form with rapidity. I have known them entirely defined within a few hours. The kiesteinic pellicle, when fully formed, has almost always a much greater degree of tenacity than the others: I have often, for purposes of microscopic examination, lifted large flakes entirely out of the urine; and when it was well defined, this was easily done: with the others it was never practicable. It seems also to be independent of putrefaction; it is not obscured for some time by the disorganization of the liquid on which it rests; and the characteristics which I have already described as accompanying its appearance are very seldom simulated.

The appearances which I have observed seem to point directly to the conclusion, that the formation of the kiesteine is unconnected with the presence of extraneous pus or flocculent mucus. I was aware that these and other animal matters might under certain modifications give rise to a scum upon the surface, the "cremor urinæ" of the older writers. This has been noticed by M. Becquerel, as especially observable in leucorrhœa; and I have observed it very frequently, not only in that disease, but in cystitis, gonorrhœa, vaginal and uterine hemorrhages, and immediately after delivery when the lochial discharge was mingled with the urine."

"In the present state of physiological chemistry, but little can be determined with regard to the nature of the kiesteine, and its very doubtful claims to be considered as a new principle. The absence of coagulation by appropriate agents indicates in a measure that neither caseum nor albumen exists in perceptible quantities; while the acid reaction up to the moment of disintegration seems opposed to the idea of its being a mere attendant upon increased quantities of pus or mucus."

"The pellicle, taken immediately from the urine on a glass plate, carefully introduced, when examined with a magnifying power of one hundred diameters, exhibited, while yet moist, a well defined series of flakes of a somewhat darkish yellow, made up apparently of minute granules. This appearance, which I at first thought to consist of minute globules of mucus or pus, was at once recognised by Dr. Goddard, as closely resembling, if not identical with, the granules of the colostrum. Having with some difficulty procured a supply of this fluid, a comparison of the two exhibited still more clearly this interesting resemblance. The granules of the kiestine were however more flattened than those of the colostrum, a change they might readily have undergone during their passage through the kidneys; but the general aspect of the two was such as to give strong evidence of their identity.*

Connected with these appearances, and sometimes obscuring them, the kiestine presented under the microscope an irregularly disposed amorphous matter, sometimes arranged in groups of granules that resembled the urate of ammonia,† and sometimes of badly marked globules, allied to those of pus or mucus, and accompanied by laminæ resembling epithelia.‡

Throughout the field of the instrument was seen, in varying numbers and distribution, a series of rectangular rhomboidal prisms, more or less distinctly marked, and strongly refracting light. The triangular prisms were also occasionally distinguished, but not in the "myriads" seen by Dr. Bird; and sometimes other crystalline forms were observed in addition to these. They all belong most probably to some of the earthy phosphates. Not only were the triangular prisms recognised to be those described by Dr. Bird, as belonging to the ammoniacal phosphate of magnesia, but by comparing them with the microscopic plates of Rayer and Vigla, and Mandl, (Etude Microscopique sur l'Uurine,) many others were discovered, coinciding with the varied forms of this prominent salt."

"In the pellicle not kiesteinic, the peculiar granular arrange-

---

* This resemblance was very striking upon comparing it with the plates of M. Mandl.

† See Mandl, Raspail, and Rayer.

‡ See plates of Rayer and Vigla, Encyclgraphie Medicale, Vol. VI.

ment noticed as so strongly resembling the colostrum, was in no case present. Amorphous darkish masses, accompanied by various crystalline forms, were seen only. Where much discoloration existed from sanguineous or local intermixture, a strong resemblance was observed to the globule of the blood."

"I cannot regard the kiestine as an *unerring* test of pregnancy. I have already shown that it is present under other conditions of the system; and even where pregnancy exists, I am not satisfied that this indication is not always observable.

I am convinced too, that the kiesteine is not always distinguishable from other pellicles which appear on the surface of the urine. At least, I am bound to say, that, in the absence of other indications, I should sometimes have found myself unable to distinguish between them. Not that they are generally liable to be confounded; but between the imperfectly developed pellicle of the one character, and the best simulation of it which is sometimes presented by others, the distinction is too slight to be satisfactory or unfailing.

But with the qualifications which these remarks imply, I have no doubt that the pellicle which has been denominated kiesteine *is among the best*, if indeed it be not *the most certain*, of the earlier indications of pregnancy. I resorted to it habitually in my diagnosis in the obstetric wards of our hospital, and with constantly increasing confidence."

"The result of my observations may be summed up in the following general conclusions:

1. That the kiesteine is not peculiar to pregnancy, but may occur whenever the lacteal elements are secreted without a free discharge at the mammæ.

2. That though sometimes obscurely developed and occasionally simulated by other pellicles, it is generally distinguishable from all others.

3. That where pregnancy is possible, the exhibition of a clearly defined kiesteinic pellicle, is one of the least equivocal proofs of that condition; and

4. That when this pellicle is not found in the more advanced stages of supposed pregnancy, the probabilities, if the female be otherwise healthy, are as 20 to 1 (81 to 4) that the prognosis is incorrect."

"Since this dissertation was submitted to the Medical Faculy of the University of Pennsylvania, two papers on this subject have appeared; one by Mr. Letheby, in the *London Medical Gazette* of Dec. 24, 1841, and an elaborate paper by Dr. Stark, in the *Edinburgh Medical and Surgical Journal*, for January of the present year.

The facts observed by Mr. Letheby accord *generally* with my own. He found 'unquestionable evidence of kiesteine in forty-eight out of fifty cases between the second and ninth month of utero-gestation,' and was unable, like myself, to 'account for its absence in the two exceptions.' In seventeen non-pregnant women he found no indication of its presence; but detected it in the urine of ten suckling women, immediately after delivery, and onwards to periods between the second and sixth months, when it disappeared. The few microscopic results which he gives coincide also with my own.

Dr. Stark's paper is devoted to the signs of pregnancy, and among the rest to the state of the urine. He refers succinctly to his own observations relative to the kiesteinic pellicle, which he supposes to be derived from the suspended sediment; and he asserts that there exists a relative proportion between these and the earthy salts which enter into the composition of the urine. His paper derives its principal interest from his researches into the character of the sediment.

In the *natural sediment* of the urine of pregnancy, he was unable to detect the presence either of albumen or caseum by acids, alkalis, or alcohol, with the aid of heat; but when he added a certain quantity of milk to the urine, both these principles were discovered readily by the aid of acids, the other tests producing no effect: when milk was added in smaller quantities, however, it was *undiscoverable by any reagent.* Being unable to refer the sediment to any of the known deposites of the urine, though his investigations had immediate reference to its distinctive characters, he employed ether to effect a separation of the *animalized matter* which he thought it might contain, and he supposes that he succeeded by this means in detecting a substance entirely different from any heretofore known.

Failing to determine its constitution by chemical agents, he resorted to the microscope. He here found that this sedimentitious matter, whether examined while yet held in solution by the recent urine, or when it had assumed the form of a deposit, or when it had been disengaged by ether, was composed of distinct transparent or 'pellucid' globules, which when in their sedimentary condition bore a striking resemblance to the caseum globule of recent milk, but which when pellucid bore an equally strong resemblance to the serous or albuminous globule.

Dr. Stark now reduced the question to very narrow bounds, by inquiring as to the identity of this sedimentary matter with albumen, caseum, fibrine, and gelatine. He thinks that its minute structure and chemical properties sufficiently distinguish it from the two first;—from albumen, because it dissolves, instead of coagulating, upon the application of heat; from caseum, because it is soluble in nitric and sulphuric acids, which exert on this principle a very contrary action. From fibrine, it has necessarily a still greater difference. There remains *gelatine;* and compared with this, the distinction was less striking. The globules of both under the microscope, were similarly formed, both were soluble in the acids and alkalies, and by the aid of heat. The only distinctive particular seems to have been the action of tannin, which, as is well known, precipitates gelatine from its solution in water. Some of the natural sediments, dissolved in boiling water, and cooled to a blood heat, were treated with a decoction of galls: a flocculent precipitate was at once produced; but instead of gelatinizing upon cooling—*it was deposited*; and instead of becoming more solid and more easily separable, upon reboiling, it again underwent solution.

Upon these grounds he attains the conclusion, which I give without comment, "that this substance is a matter *sui generis*, an elementary substance or principle, forming in some measure a connecting link between the albuminous and gelatinous elementary principles.' This substance he proposes to designate by the name of '*Gravidine*.'

It is unnecessary to say, that this discovery of a new organic principle, if confirmed by future investigation, will be a matter of great interest. I must confess, however, that the distinctive characteristics of the new substance do not seem to me very decidedly marked in the results announced by Dr. Stark; and such is the complex, and often deceptive nature of the investigations of physiological chemistry, that we have a right to wait for renewed experiments before admitting too implicitly the certainty of those he has described.

Dr. Stark considers that his experiments entirely subvert an opinion which has met with some favor regarding the theory of these appearances. Ever since the publication of Nauche's paper, the supposed presence of caseum in the urine of pregnancy has countenanced the idea entertained by Bird and others, already referred to, that the elements of the milk (not, as Dr. Stark infers, the milk itself,) might probably exist in the urine:—as, however, the matter is neither milk nor caseum, a theory based upon their presence must necessarily fall. The conclusion may be a correct one so far as the chemical analysis is concerned; and yet the connection between the kiestine pellicle and the mammary secretion may be adequately proved by other evidence. If even the Gravidine be regarded as a new organic principle, its properties are not so peculiar, nor its analogies with caseum so remote, as necessarily to imply the operation of different causes in the formation of the two.

I have already mentioned my conviction, founded on personal observations, that the unmodified caseum is not found in the urine; but the presence of the colostral appearances under the microscope, and the numerous phenomena which I have described as attending the presence of the Kiesteine leave me no room to doubt its *intimate connection with the condition of lactation.*"

